# Supplementary material for: Learning curve and surgical outcome of robotic assisted colorectal surgery with ERAS program
Source: Sci Rep. 2022 Nov 29;12:20566. doi: 10.1038/s41598-022-24665-w (PMC9709162; doi:10.1038/s41598-022-24665-w)
Supplement: Supplementary file 1 — Supplementary Information 1. [file 41598_2022_24665_MOESM1_ESM.zip › LSC Raw data-Ñ[▒K/059/OP_ñw╝╨░O▒Kñσ.pdf]

正式摘要(電子簽章：000000183807564)

請選擇...reporter

臺中榮民總醫院

[redacted]

- 手術日期：2020/01/09
- 手術醫師：VS 蔣鋒帆，CR 江世偉，R 邱俊凱，R 吳中文
- Anesthesia：GE

Diagnosis

- Pre-operative diagnosis :CA OF RECTUM 10CM FAV
- Post-operative diagnosis : Ditto.

Surgical Indication

- For curative resection

Operation

- 1. Laparoscopic low anterior resection, end-to-end anastomosis, endo-GIA 60/4.1, SDH 33mm. Bilateral doughnut: intact, Air leak test: done, no air leak.
  2. Loop ileostomy at RLQ.
  3. High ligation (+) above the left colic artery.
  4. Cut margin: proximal 15cm, distal 5cm.
  5. Take down of splenic flexure: nil.
  6. A 12mm hemovac placed in the pelvis.

Finding

- 1. A 3 x 3cm polypoid tumor at middle rectum, which invaded to the muscular layer, but didn't penetrate through the serosa. No enlarged lymph nodes at mesorectum, cT3N0M0.
  2. There were no synchronous polyps or tumor at resected specimen.
  3. There was no palpable liver metastasis, no visible peritoneal seeding.

Procedure

- General endotracheal anesthesia was induced and then the patient was placed in modified Trendelenburg lithotomy position. Preoperative antibiotics were given. A Foley catheter and nasogastric tube were placed. The abdomen was prepped and draped in standard sterile fashion. A small vertical incision was made above the umbilicus and a 12mm trocar was inserted into the peritoneal cavity by open method. Peumoperitoneum was established with CO2 inflation. Laparoscopy was inserted and then another two 5mm trocars were inserted over LLQ and RUQ of abdomen. One 12mm trocar was inserted over RLQ. The operative findings were mentioned as above. The sigmoid colon was held by grasper and the peritoneum overlying the mesentery was scored with electrocautery. The inferior mesentery artery was identified, ligated above the left colic artery with endoclips, and transected. Dissection was done above the Gerota's fascia and toward the peritoneal attachments. The colon was mobilized from retroperitoneum overlying the sacral promontory and into the pelvis. The incision was extended laterally and medially across the peritoneal reflection. The mesorectum was then mobilized. Distal descending colon and rectum were mobilized. The mesorectum was then mobilized using sharp dissection until the levator ani muscle was identified. It was determined a CDH 33mm could be accomondated and the proximal colon was divided. The rectum was divided with ENDO-GIA60/4.1. The midline wound was enlarged and the rectum was pulled out. The distal descending colon was transected and the specimen was removed from the operative field. The cap of SDH was applied at the distal end of the descending colon. Pneumoperitoneum was constructed again. End-to-end anastomosis was established with SDH 33mm. A 12mm penrose drain was placed in pelvis. One extended transverse incision at 12mm

- trocar site of RLQ. The terminal ileum was pulled out of the abdomen. A plastic bridge was placed. The fascia was closed with interrupted sutures of No.1 Vicryl. The skin were closed with interrupted 3-0 Nylon. The ileostomy was matured with 4-0 monocryl suture with nipple at proximal limb. An ostomy appliance was placed. The patient tolerated the procedure well and was brought to the POR in stable condition.
- Estimated blood loss: minimum
- 器械數量: 正確
- 紗布數量: 正確

Image

- PAINT
  - abdomen

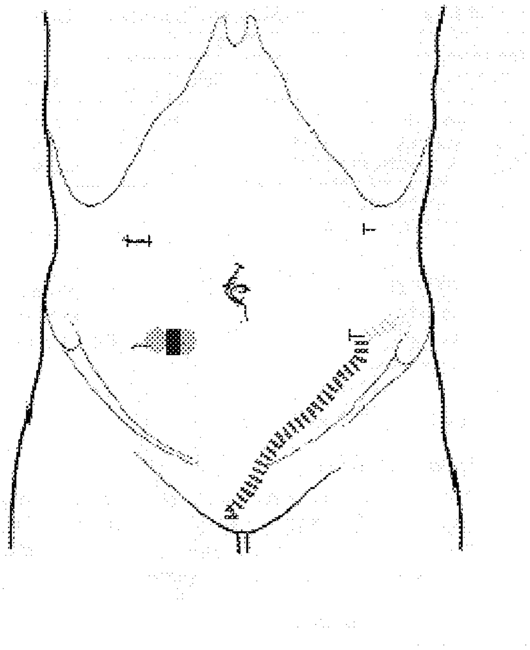

  - colon & rectum

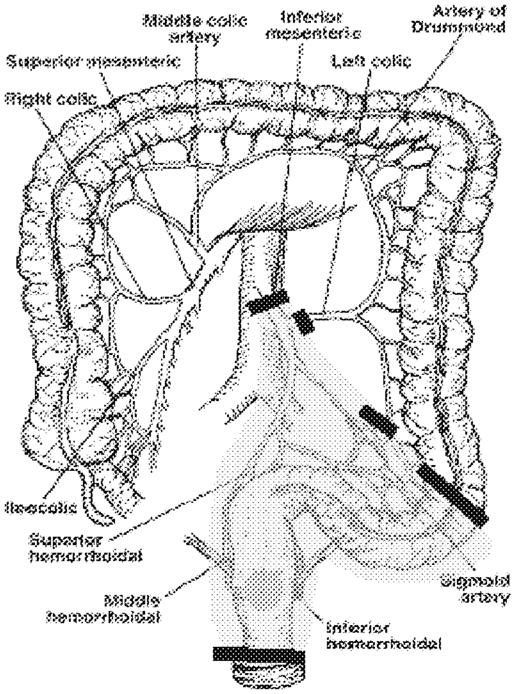

編輯主任 2062F蔣鋒帆、主治 2288K江世偉

正式摘要(電子簽章：000000183807564)

請選擇...reporter

doctorRank:B
